# New Work – Old Problem? Wie Postbürokratie die Digitalisierung erschwert

**DOI:** 10.1007/s11613-023-00811-9

**Published:** 2023-03-09

**Authors:** Lene Baumgart

**Affiliations:** Lehrstuhl für Organisations- und Verwaltungssoziologie, Universität Potsdam, August-Bebel-Str. 89, 14482 Potsdam, Deutschland

**Keywords:** Digitalisierung, Postbürokratie, Funktionale Differenzierung, Digitalization, Post-Bureaucracy, Functional Differentiation

## Abstract

Die Nutzung digitaler Kollaborationstools wird als Voraussetzung für eine postbürokratische New Work-Welt erachtet. Organisationale Digitalisierungsprojekte zur Einführung solcher Kollaborationssoftware sind selbst postbürokratisch strukturiert, d. h. sie arbeiten in crossfunktionalen und selbstorganisierten Teams. Während der Kooperation mit anderen Organisationseinheiten treten Konflikte auf, die sich dadurch verschärfen, dass sie nicht von der Hierarchie gelöst werden können, sondern im Sinne von New Work demokratisch ausgehandelt werden müssen. In der Folge bedarf es alternativer formaler Strukturen, die diese Herausforderung bewältigen.

## Abstract (en)

The use of digital collaboration tools is considered a requirement for a post-bureaucratic New Work world. Organizational digitalization projects for the introduction of such collaboration software are themselves post-bureaucratically structured, i.e., they work in cross-functional and self-organized teams. During cooperation with other organizational units, conflicts arise that are exacerbated by the fact that they cannot be resolved by the hierarchy but must be negotiated democratically in the spirit of New Work. As a result, alternative formal structures are needed to deal with this challenge.

## Zum Verhältnis von Digitalisierung und New Work

Unter dem Schlagwort „New Work“ wird neben einer Zunahme an agilen, postbürokratischen Arbeitsweisen auch die Digitalisierung von Arbeit diskutiert (Mitev et al. 2021; Weber und Thomson 2022, S. 226). Die Welt der „Neuen Arbeit“ ist eine digitalisierte Welt, in der dezentrale und netzwerkartige Strukturen herrschen, Hierarchien aufgelöst werden, Teams selbstorganisiert arbeiten und Organisationsmitglieder alle nach demselben „Purpose“ streben (vgl. Kühl 2015a, 2020). Diese neue Art der Arbeit soll auch durch den Einsatz digitaler Kollaborationstools ermöglicht werden. Der vorliegende Beitrag basiert auf dem empirischen Beispiel einer Organisationseinheit mit dem Namen *WorkingDigitally*^1^, die dafür zuständig ist, ebensolche digitalen Technologien zu entwickeln und sie in ihrer Organisation über Abteilungsgrenzen hinweg zu implementieren. Dabei fällt auf: Die Einheit ist nicht nur dafür zuständig, mithilfe der Kollaborationstools die Bedingungen für New Work zu schaffen – sie ist bereits selbst als „postbürokratische“ Struktur (vgl. Heckscher 1994) im Sinne von New Work aufgesetzt. Das heißt, sie arbeitet projektförmig, in selbst organisierten, weitgehend hierarchiefreien und crossfunktionalen Teams.

Als agile Einheit innerhalb eines deutschen Konzerns stößt *WorkingDigitally* insbesondere an ihren Grenzen zur restlichen Organisation auf Herausforderungen: Während der Einführung digitaler Technologien kommt es zu zahlreichen Kommunikationspunkten mit anderen Bereichen (u. a. IT-Security und Betriebsrat), die langwierige Verhandlungen mit einem hohem Konfliktpotenzial auslösen. Damit trifft *WorkingDigitally* auf das Urproblem aller Organisationen: ihre funktionale Differenzierung (Luhmann 2000, S. 73). Wenn Organisationen ihre Arbeit auf mehrere Abteilungen verteilen, bilden sich in diesen Einheiten „lokale Rationalitäten“ (Cyert et al. 1963) heraus, die mit den Zwecken und Herangehensweisen anderer Einheiten konfligieren (Muster und Büchner 2018, S. 256). Jede funktional differenzierte Organisation stößt auf dieses Folgeproblem, das sich verschärft in postbürokratisch strukturierten Organisationen, die auf Hierarchien verzichten und stattdessen demokratische, auf Mitbestimmung ausgerichtete Aushandlungsprozesse installieren (vgl. Eckstein und Muster 2021).

Der vorliegende Beitrag beruht demnach auf diesen zwei Thesen: (1) Vor allem Digitalisierungsinitiativen werden mit dem Problem der funktionalen Differenzierung konfrontiert, da die Digitalisierung die Gesamtheit einer Organisation betrifft (Rachlitz 2022, S. 26) oder Digitalisierungsabteilungen mit vielfältigen Querschnittsaufgaben betraut werden (Buchholz und Meyer 2022, S. 293).^2^ (2) Die in New Work immanenten Strukturen intensivieren die Folgeprobleme der funktionalen Differenzierung. Aus diesen Thesen leitet sich die für die Praxis wie für die Wissenschaft gleichermaßen relevante Frage ab, wie agile Digitalisierungsinitiativen es trotz dieser Herausforderungen schaffen, nachhaltig digitale Technologien zu implementieren. *WorkingDigitally *reagiert auf die Herausforderungen mit zwei Mechanismen: (1) mit einer crossfunktionalen Zusammenarbeit innerhalb der operativen Teams und (2) mit der Etablierung von Stellen, die explizit für die Verhandlungen an ihren internen Organisationsgrenzen zuständig sind. Damit wird das Problem produktiv gewendet: Lokale Rationalitäten werden von Beginn an offengelegt, diskursiv bearbeitet und von dafür geschultem Personal bestmöglich ausgehandelt.

Bevor diese Beobachtungen im Folgenden weiter ausgeführt werden, soll zunächst ein kurzer Abriss des aktuellen Forschungsstandes helfen, den Organisationswandel durch die Digitalisierung einzuordnen. Bei der Rekonstruktion des Forschungsstandes fällt auf, dass es bisher zwar viele soziologische Auseinandersetzungen mit der Frage gibt, wie Organisationen sich durch die Digitalisierung verändern, es aber an Studien darüber mangelt, wie Organisationen Digitalisierungsprozesse aufsetzen, mit welchen Herausforderungen sie konfrontiert sind und welche Rolle postbürokratische Strukturen dabei spielen. Dieser Lücke soll über die anschließende Rekonstruktion des Fallbeispiels *WorkingDigitally* Abhilfe geschaffen werden. Die Beschreibungen werden schließlich organisationssoziologisch eingeordnet und Lösungsvorschläge abgeleitet.

## Organisationswandel durch Digitalisierung

Der vorliegende Beitrag basiert auf einem systemtheoretischen Organisationsverständnis, das Organisationen als soziale Systeme begreift, die auf freiwilliger und revidierbarer Mitgliedschaft basieren (Luhmann 1999). Sie zeichnen sich besonders durch ihre formalen Strukturen aus, über die entschiedene Verhaltenserwartungen an die Mitglieder gestellt werden, deren Anerkennung allgemeine Mitgliedschaftsbedingung ist. Die Rigidität der Formalstruktur wird durch die informalen Strukturen ausgeglichen, unter die alle routinisierten Verhaltensweisen gefasst werden können, die nicht formal entschieden wurden.

Die Einführung digitaler Technologien und die Nutzung elektronischer Datenverarbeitungsprogramme führen zu zahlreichen Entscheidungsbedarfen und damit zu einer Aufrichtung der organisationalen Formalstruktur (u. a. Büchner 2018; Mormann 2013; Rachlitz 2022).^3^ Von Unternehmenssoftware generierte Daten oder die Kategorisierungen von Algorithmen erzeugen eine Grundlage für Anschlussentscheidungen (Büchner und Dosdall 2021; Mormann 2013). Organisationale Prozesse müssen exakt definiert und codiert werden, und die Regeln ihrer Verarbeitung müssen algorithmisiert werden, bevor sie automatisiert werden können (Wehrsig und Tacke 1992, S. 236). Für Digitalisierungsvorhaben wird häufig ein formales Projekt aufgesetzt (Mormann 2016), das mit einem Ziel und einer Befristung ausgestattet wird (Mormann und Willjes 2013). Damit wird die Gesamtorganisation entlastet, weil diese sich auf das normale Tagesgeschäft konzentrieren kann, während das Projekt quer zur Struktur liegt und parallel an der Lösungsumsetzung und Zielerreichung arbeitet (Mormann und Willjes 2013, S. 30). Sowohl innerhalb des Projekts als auch an seinen Grenzen zur Regelorganisation sind zahlreiche weitere formale Entscheidungen erforderlich: Welche Abteilungen müssen involviert werden? In welchen Bereichen soll die Software Anwendung finden? Wer ist für den Betrieb zuständig, wenn das Projekt als beendet gilt?

Mit der Zunahme digitaler Technologien und der Wirkmächtigkeit von Daten kommt es außerdem zu Verschiebungen der Machtverhältnisse (Simon et al. 2022). Es werden Stellen relevanter, die Softwareprogramme schreiben und/oder zur Verfügung stellen (Muster und Büchner 2018, S. 262), und solche, die Daten verarbeiten, einschätzen und bewerten können (Schreckling und Steiger 2017). Abteilungen, die Bedingungen an die neue Technik knüpfen können (etwa der Betriebsrat, die IT-Security oder die Rechtsabteilung), kontrollieren relevante Hebel in der Organisation (Ortmann et al. 1990, S. 151). Derartige Stellen erhalten damit – zumindest informal – mehr Macht.

Was bei diesen Beschreibungen weitgehend fehlt, ist eine Auseinandersetzung mit der Frage, wie Organisationen den Prozess ihrer Digitalisierung angehen. Erste soziologische Arbeiten dazu beobachten z. B. eine Diskrepanz zwischen den Ankündigungen von Digitalisierungsprojekten und deren schlussendlicher Implementierung (Buchholz und Meyer 2022, S. 288). Die Narrative, die in und über die Digitalisierung von Organisationen verbreitet sind, sind oftmals weitgehend von den existierenden digitalen Praktiken entkoppelt (Buchholz und Meyer 2022, S. 296). Die Studie von Simon et al. (2022) zeigt, wie Digitalisierungsprozesse entgegen vielen Annahmen nicht entlang der formalen Hierarchie von oben nach unten entschieden werden, sondern aufgrund gewachsener Mitbestimmungsstrukturen vielmehr bottom-up gestaltet und umgesetzt werden.

Solch postbürokratischen Mitbestimmungsstrukturen sind es, die in den letzten Jahrzehnten im Zuge der Agilitätsbestrebungen in Organisationen eingeführt wurden (vgl. Kühl 2015b). Diese organisationale Transformation hin zu einer agileren New Work-Welt wird fast selbstverständlich mit Digitalisierungsvorhaben gekoppelt (Muster und Büchner 2018, S. 272). Der Grund hierfür ist, dass mit der Digitalisierung eine Hoffnung auf weniger Bürokratie und mehr Rationalisierung verbunden wird (Büchner 2018, S. 333, 339; Weber und Thomson 2022, S. 232). Tatsächlich aber bestätigt sich die Rationalisierungsannahme nicht konsequent – im Gegenteil: Das Implementieren, der Umbau oder die Abschaffung digitaler Programme entpuppen sich als wesentlich langsamer und weniger elastisch als erwartet (Büchner 2018, S. 339; vgl. Trittin-Ulbrich et al. 2021, S. 11). Der Bedarf nach formalen Entscheidungen und Strukturen, der während der Digitalisierungsprozesse aufkommt, widerspricht dem Wunsch von New Work nach weniger starren Regelungen und Formalismen. Die gesteigerten Formalisierungsbedarfe werden auch beim Beispiel *WorkingDigitally *in demokratischen Mitbestimmungsstrukturen diskutiert, was sich als größte Herausforderung für das Digitalisierungsprogramm entpuppt. Der vorliegende Beitrag soll die Forschungslücke daher auf zwei Weisen schließen: Erstens wird systematisch beschrieben, wie eine Organisation eine Digitalisierungseinheit strukturell aufsetzt, wie diese operativ arbeitet und wie ihre Strukturen sich im Laufe der Zeit transformieren. Zweitens wird gezeigt, dass es die postbürokratischen Strukturen sind, die das alte Problem der funktionalen Differenzierung für das Digitalisierungsvorhaben verschärfen. Im folgenden Abschnitt wird zuerst die methodische Herangehensweise bei der Erhebung und Auswertung des Datenmaterials beschrieben und anschließend der Fall rekonstruiert.

## Fallrekonstruktion: Organisationales Subsystem *WorkingDigitally*

Die empirische Grundlage dieses Beitrags bildet eine Organisation mit einer langen bürokratischen Historie, die in einem stark digitalisierten Feld operiert. Im Rahmen eines Forschungsprojekts habe ich dort fünfzehn qualitative leitfadengestützte Interviews und vier Explorationsgespräche^4^ geführt, wobei alle Gesprächspartner:innen als Expert:innen für die organisationalen Strukturen adressiert wurden (vgl. Bogner und Menz 2002). Alle Interviews wurden via Videokonferenzplattform geführt und dauerten ca. 60 Minuten. Sie wurden aufgezeichnet, transkribiert und pseudonymisiert. Die Explorationsgespräche wurden dicht protokolliert und ebenfalls ausgewertet. Zusätzlich habe ich die Ergebnisse der Interviews und Explorationsgespräche mit den Erkenntnissen einer Dokumentenanalyse über die Formalstrukturen der Organisation trianguliert. Die Interviewpartner:innen wurden nach ihrer Funktion ausgewählt, um die Sichtweise möglichst vieler Rollen einzubeziehen. Für den hier zu analysierenden Fall waren daher insbesondere vier der fünfzehn Interviews und zwei Explorationsgespräche von Relevanz, da diese Gesprächspartner:innen als Vertreter:innen aller existierenden Funktionen und Hierarchieebenen bei *WorkingDigitally *fungierten.^5^

Die erhobenen Daten habe ich anschließend mit der qualitativen Inhaltsanalyse nach Kuckartz und Rädiker (2022, S. 129–156) ausgewertet. Mithilfe von QDA-Software konnten thematische, theoretische und in vivo-Kategorien gebildet werden. Während thematische und in vivo-Kategorien sich induktiv aus dem Material ergaben, wurden die theoretischen Kategorien deduktiv aus der Systemtheorie abgeleitet. Die Kategorien habe ich dann in einem hierarchischen Kategoriensystem geordnet, wobei die drei Hauptkategorien „Organisationsstrukturen WorkingDigitally“, „Operative Projektarbeit“ und „Herausforderungen“ in der Auswertung besondere Relevanz bekamen. Sie haben fünf bis sieben Unterkategorien, wobei die „Organisationsstrukturen“ und „Operative Projektarbeit“ deduktiv nach den Entscheidungsprämissen (Luhmann 2000) kategorisiert und die „Herausforderungen“ induktiv aus dem Material erschlossen wurden. Da die folgende Fallrekonstruktion das kombinierte Ergebnis der Dokumenten- und Gesprächsanalysen ist, wird weitgehend auf direkte Zitate von Aussagen verzichtet und diese nur vereinzelt zur Unterstützung hinzugezogen.

Die Fallbeschreibung besteht aus einer deskriptiven Rekonstruktion des Subsystems *WorkingDigitally* und einer exemplarischen Darstellung der alltäglichen operativen Arbeit. Dafür habe ich insbesondere die Aussagen der ersten beiden Hauptkategorien berücksichtigt. Die Inhalte der dritten Hauptkategorie „Herausforderungen“ werden im darauffolgenden Abschnitt illustriert.

### Funktion und Form von *WorkingDigitally*

Ein deutscher, international agierender Konzern mit ca. 100.000 Mitarbeitenden hat 2008 ein Projekt mit dem Auftrag gestartet, eine länderübergreifende Zusammenarbeit zu fördern, indem sie durch die Nutzung einer digitalen Plattform vereinfacht werden sollte. Aus dieser Plattform wurde drei Jahre später das erste „Enterprise Social Network“ (ESN) der Organisation (I1; I2). Über das ESN sollten konzernweit Informationen geteilt sowie abteilungsübergreifende Interaktion und Kollaboration im Sinne von New Work ermöglicht werden. Parallel zu diesem Projekt wurden weitere Initiativen gestartet, die sich alle mit Aktivitäten, Tools und Ideen befassten, die im Konzern für digitale Erneuerungen und agile Zusammenarbeit sorgen sollten. Da sowohl eine Steuerung als auch eine Finanzierung dieser Projekte sichergestellt werden musste, wurden 2015 die zahlreichen *befristeten* „Projekte“ zu einem zeitlich *entfristeten* „Programm“^6^ zusammengefasst (I2). Dieses Programm „*WorkingDigitally*“ ist bis heute damit beauftragt, die Kollaboration der Organisationsmitglieder zu digitalisieren, liegt als crossfunktionale Einheit quer zur Formalstruktur des Konzerns und ist in keinem Organigramm der Organisation verankert (E1). Die Programmmitglieder sind Einheiten der Regelorganisation zugeordnet und kommen aus der zentralen Abteilung *Human Resources* (*HR*) und der zentralen Abteilung *Information Technology* (*IT*).

Das Programm hat ein Managementteam, das aus den Vorstandsmitgliedern aller Bereiche des Konzerns besteht und für die Finanzierung und Schwerpunktsetzung zuständig ist (I1; I3). Darunter arbeitet ein operatives Programmteam, das sich ebenfalls aus Mitarbeiter:innen aller Vorstandsbereiche zusammensetzt und von zwei Leiter:innen aus HR und IT geführt wird. Die operative Arbeit wird von weitgehend autonomen, hierarchiefreien und crossfunktionalen Projektteams aus HR und IT durchgeführt, die von sich angeben, agile Methoden zu nutzen (E2; I4).

*WorkingDigitally *ist für drei Aufgabenbereiche zuständig: die Entwicklung oder Anschaffung neuer *Tools*, das *Enabling *der Organisationsmitglieder in der Nutzung der Tools und die Pflege einer sich darum bildenden *Community*. In den Aufgabenbereich *Tools* fallen die Identifizierung, Entwicklung, Anpassung oder Implementierung von Soft- und Hardwareprodukten, die den Organisationsalltag der Mitglieder digitalisieren sollen. Es geht hier sowohl um Chipkarten, mit denen die Angestellten sich digital ausweisen können, als auch um die Modernisierung des ESN oder die Einführung von Kommunikationstools wie Slack. Vorschläge zu Tools kommen auf drei Wegen zu *WorkingDigitally *(I4): Entweder über einen Bedarf in der Organisation, der über die Community oder das Management eingespielt wird. Die zweite Quelle sind Weiterentwicklungen seitens der Tool-Vendoren, wenn z. B. Microsoft eine neue Cloudlösung anbietet und die Organisation hierin einen Nutzen sieht. Der dritte Grund für Tooleinführungen oder -anpassungen sind Änderungen der Rechtslage z. B. zu Datenschutzthemen, die zu einer Unbrauchbarkeit von Tools führen können.

Im Zuge des *Enablings *werden Informationsveranstaltungen, Workshops und Trainings konzipiert und angeboten, die die Konzernmitglieder in der Nutzung der Tools befähigen sollen. Dabei entwickelt und vertreibt *WorkingDigitally* auch Trainings für Tools, für deren Einführung es nicht selbst zuständig war, wie für Microsoft 365 (I3). Innerhalb des dritten Aufgabenbereichs *Community* muss ein Netzwerk gebildet und gepflegt werden, in dem sich Organisationsmitglieder befinden, die sich gegenseitig bei der Nutzung der Tools unterstützen, die persönlich an der Digitalisierung der Organisation interessiert sind und daher Impulse sowie Bedarfe in das Programm spielen (I3). Diese Community kommuniziert sowohl über das ESN als auch über einen WebEx-Channel, der zum Zeitpunkt der Interviews ca. 200 fluktuierende Mitglieder aufwies. Vor der Covid-19 Pandemie gab es analoge Treffen der Community, seither treffen sich die Mitglieder per Videokonferenz. Entgegen dem initialen Gründungszweck des Programms, mithilfe des ESN die länderübergreifende Kooperation zu fördern, kommuniziert die Community bisher nur auf Deutsch. Im Laufe der Jahre, mit Zunahme der Rezeption und Relevanz digitaler Produkte und insbesondere im Zuge der Pandemie gewann das Programm an Sichtbarkeit und Wichtigkeit im Konzern, sodass eine erneute Befristung momentan nicht in Betracht gezogen wird.

### Operative Arbeit: Von der Idee zum Produkt

Für den Arbeitsmodus innerhalb des Programms gibt es keine formalisierte Blaupause, es hat sich jedoch eine Verfahrensroutine eingeschlichen: Die operativ arbeitenden Programmmitglieder erhalten über die drei oben benannten Quellen Impulse zur Implementierung eines neuen Tools oder neuer Enabling-Angebote. Wenn das Management dem neuen Produkt (Softwaretool und/oder Training) zugestimmt hat, wird ein befristetes Teilprojekt mit Projektteam aufgesetzt, das mit der Realisierung des Produkts beauftragt wird. Im Idealfall werden für jedes Tool die nötigen Enabling-Angebote gleich mit entwickelt. Ein solches Projektteam setzt sich aus folgenden Funktionen zusammen: Es gibt einen fachlichen Systemverantwortlichen, den „topic/business owner“, der die thematische Verantwortung, jedoch keinerlei disziplinarische Weisungsbefugnisse hat. Zusätzlich gibt es einen technischen Systemverantwortlichen aus der IT, den „product manager/owner“, und einen fachlichen Datenverantwortlichen. Wer welche Verantwortlichkeit erhält, entscheidet sich nach freien Kapazitäten der Programmmitglieder und nicht nach deren hierarchischen Positionen im Konzern. Weitere Rollen sind zusätzliche Projektleitungen und eine „Bridge Head“-Funktion, die von einer:m Jurist:in besetzt wird und die die Projektmitarbeitenden auf die Verhandlungen mit dem Betriebsrat vorbereitet sowie die Terminkoordination mit diesem übernimmt.

Bevor ein Tool oder Training pilotiert werden kann, muss das Projekt mit allen Stakeholdern innerhalb der Organisation kooperieren, die es für die Legitimation des Produkts braucht. Dafür werden mehrere Prozesse parallel angestoßen (I4). Zuerst braucht es eine Prüfung der Grundanforderungen seitens IT-Security und Datenschutz (I3). Zusätzlich sind direkt zu Beginn Abstimmungen mit dem Einkauf- und Lizenzmanagement sowie mit dem Konzernbetriebsrat und seinen Ausschüssen gefordert. Vor der Pilotierung muss entschieden werden, ob man das Tool als fertigen Service einkauft oder ob es in die konzerneigene Infrastruktur überführt werden kann. Wenn nach einer ersten Pilotierungsphase positive Rückmeldungen seitens User und Stakeholder kommen, kann sich das Projektteam mit der konkreten Einführung befassen (I4). Softwaretools und Trainings müssen verschiedene Assessments in den Compliance-Bereichen und in der Rechtsabteilung durchlaufen, es bedarf Barrierefreiheitsgutachten und Ergonomiegutachten (I1). Anschließend werden Betriebs- und Serviceketten aufgebaut, und damit wird sichergestellt, dass es zuständige Ansprechpartner:innen in der Organisation gibt, falls ein Tool mal nicht funktionieren sollte. Wenn alle Abstimmungen durchlaufen sind und am Ende ein von allen Seiten legitimierter Kompromiss steht, kann das Produkt final eingeführt und nutzbar gemacht werden. Die Dauer eines solchen Teilprojekts kann zwischen wenigen Monaten und mehreren Jahren variieren, je nach Dringlichkeit und Relevanz des Themas (I2).

Bereits an dieser kurzen Fallrekonstruktion lässt sich die Aufrichtung der Formalstruktur beobachten, da das Digitalisierungsvorhaben zahlreiche Entscheidungen und Regelungen erfordert. Dies bestätigt auch die Erkenntnisse von Simon et al. (2022), dass die Entscheidung von Organisationen, ob ein digitales Tool tatsächlich pilotiert wird, maßgeblich von niedrigen Hierarchieebenen und den Endnutzer:innen abhängt, die Mehrwert und Vorteil des Produkts bewerten. Gleichzeitig wird deutlich, dass die Digitalisierungseinheit nicht nur selbst postbürokratisch aufgestellt wurde, sondern auch damit beauftragt ist, mithilfe der digitalen Kollaborationstools New Work in der Organisation zu etablieren. Das geschieht an den Grenzstellen zur restlichen Organisation, was in der Herausforderung mündet, dass konfligierende lokale Rationalitäten aufeinandertreffen, die ausgehandelt werden müssen. Im folgenden Abschnitt werden diese Herausforderungen näher in den Blick genommen.

### Wie Postbürokratie die Digitalisierung erschwert

Das systemtheoretische Organisationsverständnis ermöglicht es, *WorkingDigitally* als ein Subsystem innerhalb der Organisation zu begreifen, das einerseits von seiner organisationalen Umwelt beeinflusst wird und andererseits auf sie einwirkt (vgl. Luhmann 1999). Insbesondere an diesen Grenzstellen zu den anderen Stakeholdern in der Organisation sieht sich die agile Einheit regelmäßig mit Herausforderungen konfrontiert. Von allen Interviewpartner:innen wird insbesondere die *Abstimmung mit dem Betriebsrat* als größte Hürde genannt, da dieser strenge und spezifische Anforderungen an die neuen Tools und Trainings hat. Er sorgt außerdem für eine Verlangsamung des Prozesses, da er mehrere Ausschüsse hat, die alle ihr Einverständnis geben müssen und zu verschiedenen Zeitpunkten tagen (I3). So kommt es z. B. dazu, dass statt der geplanten vier Termine in acht Monaten zehn Termine in 20 Monaten gebraucht werden (I2; I3). Das ist darauf zurückzuführen, dass in den Verhandlungen Komplexitäten aufgedeckt werden, die aus einer einzigen Perspektive nicht ersichtlich waren:

„Wir haben jetzt auch in den Verhandlungen festgestellt, dass es Komplexitäten hat, […] wo jede so eine Perspektive drauf hatte, was uns dann in Diskussionen viel Zeit gekostet hat, wir dann tatsächlich auch tagelang drüber geredet haben und dann festgestellt haben, okay, es ist wieder ein Verhandlungstag vergangen, wann ist denn der nächste? Und bei einer Runde mit, da sind wir 14 Leute in Summe, da auch wieder den nächsten Folgetermin zu finden, das ist nicht so einfach.“ (I2)

Ein Thema, das zum Zeitpunkt der Interviews viel diskutiert wurde, war die Nutzung von Tools, die der Konzern von extern einkauft. *WorkingDigitally* wollte z. B. die Kommunikationsplattform *Slack *einführen, um die E‑Mail-Kommunikation innerhalb des Konzerns weitgehend zu ersetzen. Zum Datenschutz der Mitarbeitenden hat der Betriebsrat in den Verhandlungen durchgesetzt, dass deren Chats jede Nacht zentral von der IT gelöscht werden (I1). Damit erfüllte Slack nicht seine ursprünglich angedachte Funktion und wurde folglich kaum genutzt. Mit steigendem Druck auf den Betriebsrat hat dieser den Rhythmus der Löschung vergrößert, sodass die Chats heute nur noch einmal im Monat gelöscht werden.

Das Beispiel macht deutlich, dass es zu einem doppelten Komplexitätsschock für das Digitalisierungsprogramm kommt: Auf organisationaler Ebene wird es mit Problemen und Konflikten an den Grenzen zum Konzern konfrontiert. Auf Digitalisierungsebene gehen mit der Einführung der digitalen Technologien besonders komplexe und abteilungsübergreifende formale Anforderungen einher. Dass es zu solchen Herausforderungen kommt, ist keine genuine Neuheit von New Work oder der Digitalisierung, sondern ist erst einmal als Folgeproblem der funktionalen Differenzierung zu fassen. Trotzdem lässt sich mithilfe des Beispiels behaupten, dass es die demokratischen Aushandlungsprozesse und bewusst vermiedenen hierarchischen Strukturen sind, die die verschiedenen lokalen Rationalitäten intensiver und regelmäßiger einander gegenüberstellen, die Konflikte in die Länge ziehen und die für Verantwortungsdiffusion sorgen (hierzu auch Eckstein und Muster 2021). Der Konzern hat einen Weg gefunden, mit diesem Folgeproblem umzugehen. Dieser wird im Folgenden vorgestellt.

## Altes Problem, neu gelöst?

Die Herausforderung, mit der *WorkingDigitally *sich konfrontiert sieht, ist die funktionale Differenzierung als das Urproblem jeder größeren Organisation. Jedes wachsende soziale System ist ab einer gewissen Größe darauf angewiesen, sich in verschiedene Subsysteme mit je eigenen Zuständigkeiten, Informationskanälen und Arbeitsweisen zu untergliedern, um auf die internen Ansprüche und die Anforderungen an ihren Umweltgrenzen zu reagieren (Luhmann 1999, S. 73). In jedem dieser Subsysteme bilden sich eigene Denkstrukturen und Überzeugungen (Muster und Büchner 2018, S. 256), das heißt „lokale Rationalitäten“ (Cyert et al. 1963) heraus. In der direkten Gegenüberstellung mit anderen Subsystemen sind diese lokalen Rationalitäten^7^ oftmals widersprüchlich, denn sie verfolgen je eigene Zwecke, weisen Handlungen auf, die sich nicht gradlinig auf den Gesamtzweck des Systems zurückführen lassen und verschiedene Systemprobleme „teils günstig, teils nachteilig berühren“ (Luhmann 1999, S. 75 f.). Damit wird die funktionale Differenzierung zur Hauptquelle für Konflikte in Organisationen: „Alle Arbeitsteilung in spezialisierten Großsystemen tendiert in dieser Weise zu innerem Zerfall, zur Auflösung in enge Identifikationen, die das Gesamtsystem zu sprengen drohen“ (Luhmann 1999, S. 83).

Gleichzeitig ist es die funktionale Differenzierung, die ein komplexes System in die Lage versetzt, mehrere Aufgaben gleichzeitig zu bearbeiten und so seine Leistungen zu steigern (Luhmann 1999, S. 73). Die Interessenskonflikte und Inkohärenzen sind keine organisationalen Dysfunktionen, sondern „der Tribut, den eine Organisation zahlen muss, wenn sie bestehen will“ (Crozier und Friedberg 1979, S. 57). Auf diese Weise werden die Subsysteme entlastet, denn sie können ihren Zuständigkeiten weitgehend rücksichtslos nachgehen ungeachtet dessen, was in anderen Einheiten passiert (Luhmann 1999, S. 77). Ihnen fällt ein gewisser Primat einer spezifischen Funktion zu, doch erst im Zusammenspiel mit weiteren Subsystemen kann Erfolg bewirkt werden – auch wenn der Erfolg von den einzelnen Einheiten nur als Kompromiss wahrgenommen wird. Der organisationale Mechanismus, der Konflikte maßgeblich entschärft und in Organisationen immer mitläuft, sind formale Mitgliedschaftserwartungen. Diese regulieren Ein- und Austritt von Organisationsmitgliedern und legen allgemeine (verbale) Verhaltensbedingungen, Anpassungsnotwendigkeiten sowie eine pauschale Orientierung zum Gesamtsystem fest (Luhmann 1999, S. 81).^8^ Es gehört zur formalen Mitgliedsrolle, die Arbeit anderer Subsysteme anzuerkennen und Missmut zumindest nicht öffentlich zu äußern. So kann von allen Mitgliedern von *WorkingDigitally* formal erwartet werden, dass sie ein Verständnis dafür haben, dass IT-Sicherheit, Datenschutz und andere rechtliche Bedingungen eingehalten werden müssen und dass auch die Bedenken des Betriebsrats akzeptiert werden. Damit bedingen Differenzierung und formale Generalisierung eines Systems sich wechselseitig (Luhmann 1999, S. 82).

*WorkingDigitally *nutzt zwei formale Strukturen, um dieser Herausforderung entgegenzutreten: Die erste Struktur ist die Schaffung der Grenzstelle „Bridge Head“, die als Jurist:in die lokalen Rationalitäten beider Einheiten nachvollziehen kann und explizit dafür zuständig ist, die Programmmitglieder auf die Verhandlungen mit dem Betriebsrat vorzubereiten:

„Sie weiß, welche Ansichten die Betriebsräte haben [und] bringt sie dann mit rein, sodass man das quasi auch erlebt hat und weiß, was passieren kann. Und dass man mal so ein Gefühl bekommt, was sind so die kritischen Punkte und wo legt der Betriebsrat den Finger in die Wunde, dass man da schon vorbereitet ist und auch Antworten vorbereiten kann.“ (I2)

Wie es der Name schon impliziert, baut diese Stelle eine Brücke zwischen den zwei widersprüchlichen Sichtweisen der Subsysteme, sie fungiert als eine Art Übersetzerin beider Perspektiven und hat damit eine integrative Funktion für das Gesamtsystem.

Die zweite Struktur, die geschaffen wurde und die gerade in postbürokratischen Konzepten vielfach vorgesehen ist (Heckscher 1994), ist die crossfunktionale Zusammenarbeit von HR und IT samt deren netzwerkartigen Kommunikationskanälen. Dadurch, dass das Programm genuin mit HR- und IT-Angestellten besetzt ist, sind sie auf Kollaboration und Verständnis für die jeweils andere Seite angewiesen. Gemeinsam müssen sie nach der optimalen Lösung suchen, die für die jeweilige Einheit zwar nur ein Kompromiss darstellt, für das Gesamtsystem aber das produktivste Ergebnis ist, das ohne die Auseinandersetzung nicht zustande kommen kann. Die Mitglieder von *WorkingDigitally *haben diese Kooperation als besonders gut funktionierend hervorgehoben:

„Also, was gut läuft, was wir auch, glaube ich, manchmal außer Acht lassen, ist, dass diese grundsätzliche –, dieses grundsätzliche Bekenntnis von verschiedenen Bereichen, crossfunktional an einem Thema auch inhaltlich zusammenzuarbeiten, dass das grundsätzlich schon gut läuft. […] Das merken wir im Übrigen auch eigentlich nur dann, wenn wir mit anderen Firmen sprechen, wie die ihre Tools einführen. Und dann sagen die zu uns, ach echt, das funktioniert bei euch, boa, krass. Wie, aber erst seit kurzem? Nein, schon so und so viele Jahre. Ah ja, okay, das wäre bei uns unvorstellbar.“ (I4)

Eine Herausforderung solcher crossfunktionalen Kooperationen, die weitgehend ohne formale Interaktionsregeln auskommen, ist – und das gilt ganz allgemein für Konzepte der New Work Bewegung –, dass die individuelle Person hinter der Mitgliedsrolle an Bedeutung gewinnt (Kühl 2015a). Die Organisation ist damit darauf angewiesen, dass die Teammitglieder erfolgreich kollaborieren. Dadurch tritt die Relevanz der individuellen Person an die Stelle anderer funktional äquivalenter Strukturen, die von Personen entlasten, wie z. B. Konditionalprogramme oder Hierarchien.

Es zeichnet sich ein Spannungsfeld ab: Einerseits kommt es gerade an den Grenzen zur Mutterorganisation zu Herausforderungen für die agile Einheit. Andererseits ist es diese Mutterorganisation, die durch die Etablierung formaler Strukturen in der Lage ist, die Probleme zu entschärfen. Der folgende Abschnitt bietet einen Vorschlag, was aus diesem Spannungsfeld für die Praxis abgeleitet werden kann.

## Die produktive Wendung organisationaler Probleme

Obwohl Digitalisierungsvorhaben offensichtlich einen immensen Bedarf an Entscheidungen und formalen Strukturen auslösen, wird mit der Digitalisierung eine flexiblere, entbürokratisierte und entformalisierte neue Arbeitswelt verknüpft. Das Fallbeispiel hat dies deutlich gemacht, indem das organisationale Digitalisierungsprogramm nicht nur selbst crossfunktional und agil aufgestellt wurde, sondern auch die Funktion erfüllen soll, mithilfe der eingeführten digitalen Technologien New Work zu etablieren. Dabei wurde dem Programm die funktionale Differenzierung der Mutterorganisation zum Verhängnis, die verschiedene lokale Rationalitäten produziert und damit Konflikte zwischen Organisationseinheiten provoziert. Das ist kein neues Organisationsproblem, doch im hier aufgeführten Beispiel scheint die Kombination aus Postbürokratie und Digitalisierung das Folgeproblem der funktionalen Differenzierung verschärft und einen doppelten Komplexitätsschock ausgelöst zu haben: Da Digitalisierungsprojekte mit einer die gesamte Organisation betreffenden Querschnittsaufgabe belegt sind, potenzieren sich die Schnittstellen zu anderen Organisationseinheiten. Da die Organisation aber auf die in postbürokratischen Konzepten immanenten demokratischen, konsentorientierten Abstimmungen setzt, werden die Konfliktpotenziale erhöht, die in bürokratischen und hierarchisierten Organisationen mit einem Durchregieren der Spitze weitgehend vermieden werden könnten.

Zugleich sind es die gemeinsamen Kompromisslösungen verschiedener Subsysteme, die gewährleisten, dass alle Umweltanforderungen bedacht werden. Die Organisation ist also darauf angewiesen, das Problem anders produktiv für sich zu wenden: Durch die Zusammenstellung crossfunktionaler Teams werden die Arbeitsteilungen punktuell gebrochen und Synergien wie Widersprüche von Beginn an diskursiv bearbeitet. Damit werden die elementaren Grundanforderungen an die Technologien bestmöglich sichergestellt. Zusätzlich ist es der formale Einsatz der Bridge Head-Stelle und ihre übersetzende sowie integrative Funktion, die die Kollaboration mit anderen Organisationseinheiten und damit einhergehende Konflikte entlastet. Die formalen Mitgliedschaftsbedingungen, über die ein angemessenes verbales Verhalten der Mitglieder erwartet werden kann, laufen hierbei als Grundvoraussetzung mit. Es sind diese zur bürokratischen, hierarchisierten Organisation funktionalen Äquivalente, die als Handlungsempfehlungen aus dem hier vorgestellten Fall abgeleitet werden können. Denn es lässt sich vermuten, dass auch andere Organisationen mit dem durch New Work ausgelösten Old Problem konfrontiert werden.

## Footnotes

1 Der Originalname des Programms wurde zur Wahrung der Anonymität durch dieses Pseudonym ersetzt.

2 Unter der Digitalisierung von Organisationen wird im Anschluss an Husted und Plessner (2020, S. 7) „the use of digital technology to change organizational processes and practices“ verstanden. Da die projektförmige Implementierung von Digitaltechnologien in ihrer Organisation die Kernaufgabe von *WorkingDigitally *ist, soll diese Arbeitsdefinition für den Beitrag genügen.

3 Mit einer Veränderung der Formalstruktur kommt es auch zu einer Zunahme oder Ausdifferenzierung informaler Handlungsweisen (Funken und Schulz-Schaeffer 2008). Da solche „informalen Workarounds“ für diesen Beitrag aber nicht weiter von Interesse sind, kann hier nur auf weiterführende Literatur dazu verwiesen werden (z. B. Büchner 2018; Simon et al. 2022; Trittin-Ulbrich et al. 2021).

4 Die Interviews werden in den folgenden Abschnitten mit „I“, die Explorationsgespräche mit „E“ abgekürzt. Um Rückschlüsse auf die jeweiligen Personen zu verhindern, wurden die Dokumentformen wahllos durchnummeriert.

5 Aus Datenschutzgründen können keine konkreteren Informationen der Organisation und ihrer Mitglieder offengelegt werden.

6 Das sind die Begrifflichkeiten, die von der Organisation selbst gewählt wurden.

7 Auf Seiten *WorkingDigitally*s etwa die Implementierung der digitalen Tools, sodass deren Funktionalität optimal gewährleistet ist; von Seiten des Betriebsrats die Wahrung potenzieller Mitgliederinteressen und deren Datenschutz. Das Beispiel *Slack *hat gezeigt, dass die Anforderungen so konträr waren, dass mit der Kompromisslösung die Funktionalität des Tools obsolet wurde.

8 Auf andere, insbesondere informale Möglichkeiten der Konfliktlösung geht Luhmann (1999, S. 245) ein.
